# 2-Methyl-*N*-(3-methyl­benzo­yl)benzene­sulfonamide

**DOI:** 10.1107/S1600536810002254

**Published:** 2010-01-23

**Authors:** B. Thimme Gowda, Sabine Foro, P. A. Suchetan, Hartmut Fuess

**Affiliations:** a Department of Chemistry, Mangalore University, Mangalagangotri 574 199, Mangalore, India; b Institute of Materials Science, Darmstadt University of Technology, Petersenstrasse 23, D-64287 Darmstadt, Germany

## Abstract

In the title compound, C_15_H_15_NO_3_S, the sulfonyl and amide-bound benzene rings are oriented at dihedral angles of 83.1 (1) and 22.5 (3)°, respectively, with the almost planar S—N—C=O segment (r.m.s. deviation = 0.003 Å). The dihedral angle between the two benzene rings is 74.8 (1)°. In the crystal structure, pairs of mol­ecules are linked into centrosymmetric dimers by pairs of N—H⋯O hydrogen bonds.

## Related literature

For background literature and similar structures, see: Gowda *et al.* (2009**a* ▶,b* ▶); Suchetan *et al.* (2010 ▶).
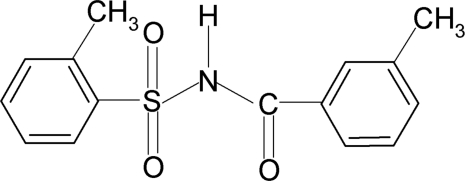


## Experimental

### 

#### Crystal data

C_15_H_15_NO_3_S
*M* *_r_* = 289.34
Monoclinic, 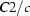

*a* = 18.023 (4) Å
*b* = 12.045 (3) Å
*c* = 17.335 (4) Å
β = 127.67 (1)°
*V* = 2978.7 (12) Å^3^
*Z* = 8
Cu *K*α radiation
μ = 1.99 mm^−1^
*T* = 299 K
0.30 × 0.18 × 0.18 mm

#### Data collection

Enraf–Nonius CAD-4 diffractometer
10158 measured reflections
2654 independent reflections
2219 reflections with *I* > 2σ(*I*)
*R*_int_ = 0.072
3 standard reflections every 120 min  intensity decay: 1.5%

#### Refinement

*R*[*F* ^2^ > 2σ(*F* ^2^)] = 0.058
*wR*(*F* ^2^) = 0.176
*S* = 1.08
2654 reflections
187 parameters
1 restraint
H atoms treated by a mixture of independent and constrained refinement
Δρ_max_ = 0.41 e Å^−3^
Δρ_min_ = −0.28 e Å^−3^

### 

Data collection: *CAD-4-PC* (Enraf–Nonius, 1996 ▶); cell refinement: *CAD-4-PC*; data reduction: *REDU4* (Stoe & Cie, 1987 ▶); program(s) used to solve structure: *SHELXS97* (Sheldrick, 2008 ▶); program(s) used to refine structure: *SHELXL97* (Sheldrick, 2008 ▶); molecular graphics: *PLATON* (Spek, 2009 ▶); software used to prepare material for publication: *SHELXL97*.

## Supplementary Material

Crystal structure: contains datablocks I, global. DOI: 10.1107/S1600536810002254/ci5020sup1.cif

Structure factors: contains datablocks I. DOI: 10.1107/S1600536810002254/ci5020Isup2.hkl

Additional supplementary materials:  crystallographic information; 3D view; checkCIF report

## Figures and Tables

**Table 1:** Hydrogen-bond geometry (Å, °)

*D*—H⋯*A*	*D*—H	H⋯*A*	*D*⋯*A*	*D*—H⋯*A*
N1—H1*N*⋯O1^i^	0.83 (2)	2.06 (2)	2.884 (4)	179 (4)

Symmetry code: (i) 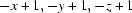.

**Fig. 1.**
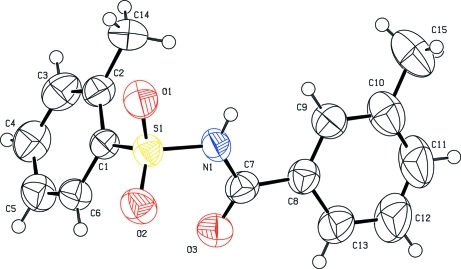
Molecular structure of the title compound, showing the atom-labelling scheme. Displacement ellipsoids are drawn at the 50% probability level.

**Fig. 2.**
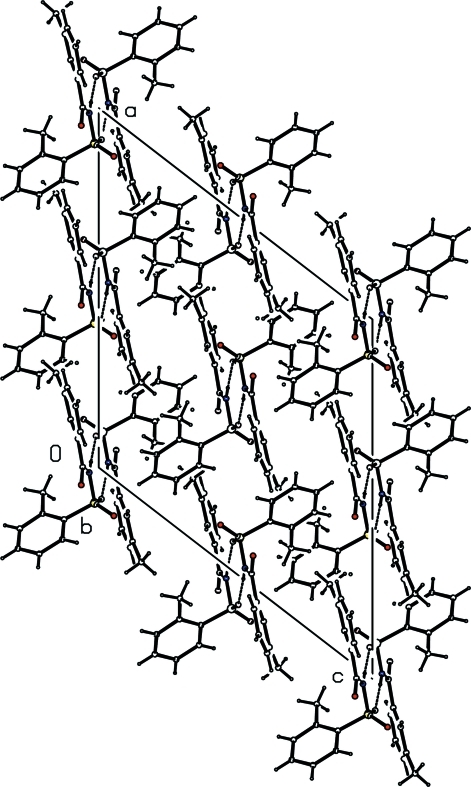
Molecular packing in the title compound. Hydrogen bonds are shown as dashed lines.

## supplementary crystallographic information

### Comment

Diaryl acylsulfonamides are known as potent antitumor agents against a broad spectrum of human tumor xenografts in nude mice. As part of a study of the effect of ring and the side chain substituents on the crystal structures of *N*-aromatic sulfonamides (Gowda *et al.*, 2009*a,b*; Suchetan *et al.*, 2010), in the present work, the structure of *N*-(3-methylbenzoyl)-2-methylbenzenesulfonamide (I) has been determined (Fig.1). The conformation of the N—H bond in the C—SO_2_—NH—C(O) segment of the structure is *anti* to the C═O bond, similar to that observed in *N*-(benzoyl)benzenesulfonamide (II) (Gowda *et al.*, 2009*a*) and *N*-(3-chlorobenzoyl)- benzenesulfonamide (III)(Gowda *et al.*, 2009*b*). The molecule is twisted at the S atom with a dihedral angle of 83.1 (1)° between the sulfonyl-bound benzene ring and the S-NH-C═O segment, compared to the values of 86.5 (1) in (II) and 89.9 (1)° in (III). Furthermore, the dihedral angle between the two benzene rings is 74.8 (1)° in (I) and 80.3(0.1) in (II) and 87.5 (1)° in (III).

The molecules are linked into dimers by N—H···O(S) hydrogen bonds (Table 1 and Fig. 2).

### Experimental

The title compound was prepared by refluxing a mixture of 3-methylbenzoic acid, 2-methylbenzenesulfonamide and phosphorous oxy chloride for 5 h on a water bath. The reaction mixture was cooled and poured into ice cold water. The resulting solid, *N*-(3-methylbenzoyl)2-methylbenzenesulfonamide, was separated, washed thoroughly with water and then dissolved in sodium bicarbonate solution. The compound was later reprecipitated by acidifying the filtered solution with dilute HCl. The filtered and dried compound was recrystallized to the constant melting point. Prism like colourless single crystals of the title compound were grown by slow evaporation of its toluene solution at room temperature.

### Refinement

The H atom of the NH group was located in a difference map and its positional parameters were refined with the N-H distance restrained to 0.86 (3) Å. The remaining H atoms were positioned with idealized geometry and refined using a riding model with C–H = 0.93–0.96 Å. All H atoms were refined with isotropic displacement parameters (set to 1.2 times of the *U*_eq_ of the parent atom).

### Crystal data

**Table:**

C_15_H_15_NO_3_S	*F*(000) = 1216
*M**_r_* = 289.34	*D*_x_ = 1.290 Mg m^−^^3^
Monoclinic, *C*2/*c*	Cu *K*α radiation, λ = 1.54180 Å
Hall symbol: -C 2yc	Cell parameters from 25 reflections
*a* = 18.023 (4) Å	θ = 4.8–20.5°
*b* = 12.045 (3) Å	µ = 1.99 mm^−^^1^
*c* = 17.335 (4) Å	*T* = 299 K
β = 127.67 (1)°	Prism, colourless
*V* = 2978.7 (12) Å^3^	0.30 × 0.18 × 0.18 mm
*Z* = 8	

### Data collection

**Table:**

Enraf–Nonius CAD-4 diffractometer	*R*_int_ = 0.072
Radiation source: fine-focus sealed tube	θ_max_ = 67.0°, θ_min_ = 4.6°
graphite	*h* = −21→21
ω/2θ scans	*k* = −14→14
10158 measured reflections	*l* = −20→20
2654 independent reflections	3 standard reflections every 120 min
2219 reflections with *I* > 2σ(*I*)	intensity decay: 1.5%

### Refinement

**Table:**

Refinement on *F*^2^	Secondary atom site location: difference Fourier map
Least-squares matrix: full	Hydrogen site location: inferred from neighbouring sites
*R*[*F*^2^ > 2σ(*F*^2^)] = 0.058	H atoms treated by a mixture of independent and constrained refinement
*wR*(*F*^2^) = 0.176	*w* = 1/[σ^2^(*F*_o_^2^) + (0.0835*P*)^2^ + 3.6838*P*] where *P* = (*F*_o_^2^ + 2*F*_c_^2^)/3
*S* = 1.08	(Δ/σ)_max_ = 0.007
2654 reflections	Δρ_max_ = 0.41 e Å^−^^3^
187 parameters	Δρ_min_ = −0.28 e Å^−^^3^
1 restraint	Extinction correction: *SHELXL97* (Sheldrick, 2008), Fc^*^=kFc[1+0.001xFc^2^λ^3^/sin(2θ)]^-1/4^
Primary atom site location: structure-invariant direct methods	Extinction coefficient: 0.0016 (2)

### Special details

**Table:**

Geometry. All e.s.d.'s (except the e.s.d. in the dihedral angle between two l.s. planes) are estimated using the full covariance matrix. The cell e.s.d.'s are taken into account individually in the estimation of e.s.d.'s in distances, angles and torsion angles; correlations between e.s.d.'s in cell parameters are only used when they are defined by crystal symmetry. An approximate (isotropic) treatment of cell e.s.d.'s is used for estimating e.s.d.'s involving l.s. planes.
Refinement. Refinement of *F*^2^ against ALL reflections. The weighted *R*-factor *wR* and goodness of fit *S* are based on *F*^2^, conventional *R*-factors *R* are based on *F*, with *F* set to zero for negative *F*^2^. The threshold expression of *F*^2^ > σ(*F*^2^) is used only for calculating *R*-factors(gt) *etc*. and is not relevant to the choice of reflections for refinement. *R*-factors based on *F*^2^ are statistically about twice as large as those based on *F*, and *R*- factors based on ALL data will be even larger.

### Fractional atomic coordinates and isotropic or equivalent isotropic displacement parameters (Å^2^)

**Table:**

	*x*	*y*	*z*	*U*_iso_*/*U*_eq_	
S1	0.39278 (5)	0.37414 (6)	0.48527 (5)	0.0536 (3)	
O1	0.41777 (17)	0.48868 (19)	0.50971 (18)	0.0718 (7)	
O2	0.39810 (16)	0.3050 (2)	0.55495 (15)	0.0708 (7)	
O3	0.41415 (18)	0.15139 (19)	0.4361 (2)	0.0767 (7)	
N1	0.46572 (18)	0.3289 (2)	0.4653 (2)	0.0586 (7)	
H1N	0.499 (2)	0.381 (2)	0.472 (3)	0.070*	
C1	0.2810 (2)	0.3633 (2)	0.3733 (2)	0.0499 (7)	
C2	0.2518 (2)	0.4330 (2)	0.2951 (2)	0.0592 (8)	
C3	0.1604 (3)	0.4177 (3)	0.2116 (3)	0.0777 (10)	
H3	0.1381	0.4625	0.1578	0.093*	
C4	0.1016 (3)	0.3391 (3)	0.2053 (3)	0.0773 (10)	
H4	0.0407	0.3319	0.1480	0.093*	
C5	0.1322 (2)	0.2713 (3)	0.2829 (3)	0.0715 (9)	
H5	0.0924	0.2182	0.2787	0.086*	
C6	0.2223 (2)	0.2827 (3)	0.3672 (2)	0.0590 (8)	
H6	0.2440	0.2364	0.4200	0.071*	
C7	0.4670 (2)	0.2184 (3)	0.4415 (2)	0.0600 (8)	
C8	0.5356 (2)	0.1891 (3)	0.4240 (2)	0.0605 (8)	
C9	0.5736 (2)	0.2662 (3)	0.3972 (2)	0.0674 (9)	
H9	0.5589	0.3411	0.3933	0.081*	
C10	0.6332 (3)	0.2327 (4)	0.3764 (3)	0.0878 (12)	
C11	0.6549 (4)	0.1236 (5)	0.3850 (5)	0.1154 (19)	
H11	0.6935	0.1000	0.3695	0.138*	
C12	0.6215 (4)	0.0456 (4)	0.4160 (5)	0.1150 (18)	
H12	0.6407	−0.0280	0.4251	0.138*	
C13	0.5602 (3)	0.0787 (3)	0.4328 (3)	0.0810 (11)	
H13	0.5348	0.0266	0.4503	0.097*	
C14	0.3103 (3)	0.5223 (3)	0.2966 (3)	0.0810 (11)	
H14A	0.3714	0.4934	0.3237	0.097*	
H14B	0.3161	0.5831	0.3357	0.097*	
H14C	0.2809	0.5476	0.2313	0.097*	
C15	0.6712 (4)	0.3156 (5)	0.3441 (4)	0.1154 (17)	
H15A	0.7328	0.3381	0.3991	0.138*	
H15B	0.6308	0.3793	0.3168	0.138*	
H15C	0.6744	0.2826	0.2958	0.138*	

### Atomic displacement parameters (Å^2^)

**Table:**

	*U*^11^	*U*^22^	*U*^33^	*U*^12^	*U*^13^	*U*^23^
S1	0.0517 (5)	0.0539 (5)	0.0577 (5)	−0.0039 (3)	0.0348 (4)	−0.0060 (3)
O1	0.0712 (14)	0.0582 (13)	0.0930 (16)	−0.0136 (11)	0.0538 (14)	−0.0249 (12)
O2	0.0653 (14)	0.0888 (17)	0.0578 (12)	−0.0003 (12)	0.0374 (11)	0.0127 (11)
O3	0.0791 (16)	0.0542 (13)	0.1081 (19)	−0.0074 (11)	0.0629 (15)	−0.0032 (12)
N1	0.0540 (15)	0.0563 (15)	0.0753 (16)	−0.0050 (11)	0.0445 (13)	−0.0054 (13)
C1	0.0525 (16)	0.0454 (15)	0.0561 (15)	0.0004 (12)	0.0353 (14)	−0.0020 (12)
C2	0.0656 (19)	0.0518 (16)	0.0650 (18)	0.0056 (14)	0.0423 (16)	0.0049 (14)
C3	0.081 (2)	0.079 (2)	0.0598 (19)	0.013 (2)	0.0361 (19)	0.0094 (17)
C4	0.061 (2)	0.081 (2)	0.064 (2)	−0.0008 (18)	0.0243 (17)	−0.0085 (18)
C5	0.0584 (19)	0.068 (2)	0.080 (2)	−0.0135 (16)	0.0380 (18)	−0.0138 (18)
C6	0.0602 (18)	0.0550 (17)	0.0639 (18)	−0.0037 (14)	0.0391 (15)	−0.0003 (14)
C7	0.0608 (18)	0.0506 (17)	0.0637 (18)	0.0005 (14)	0.0355 (16)	−0.0008 (14)
C8	0.0585 (17)	0.0582 (18)	0.0635 (18)	0.0009 (14)	0.0367 (15)	−0.0061 (14)
C9	0.075 (2)	0.0611 (19)	0.074 (2)	−0.0045 (16)	0.0503 (19)	−0.0077 (16)
C10	0.090 (3)	0.103 (3)	0.092 (3)	−0.013 (2)	0.067 (2)	−0.021 (2)
C11	0.116 (4)	0.110 (4)	0.173 (5)	−0.014 (3)	0.115 (4)	−0.047 (4)
C12	0.118 (4)	0.086 (3)	0.170 (5)	0.006 (3)	0.103 (4)	−0.031 (3)
C13	0.084 (3)	0.057 (2)	0.107 (3)	0.0001 (18)	0.062 (2)	−0.0099 (19)
C14	0.093 (3)	0.067 (2)	0.095 (3)	0.0019 (19)	0.063 (2)	0.019 (2)
C15	0.120 (4)	0.144 (5)	0.127 (4)	−0.023 (3)	0.099 (4)	−0.009 (3)

### Geometric parameters (Å, °)

**Table:**

S1—O2	1.421 (2)	C7—C8	1.484 (4)
S1—O1	1.433 (2)	C8—C13	1.381 (5)
S1—N1	1.643 (3)	C8—C9	1.391 (5)
S1—C1	1.750 (3)	C9—C10	1.386 (5)
O3—C7	1.209 (4)	C9—H9	0.93
N1—C7	1.398 (4)	C10—C11	1.353 (7)
N1—H1N	0.83 (2)	C10—C15	1.499 (6)
C1—C6	1.392 (4)	C11—C12	1.387 (7)
C1—C2	1.396 (4)	C11—H11	0.93
C2—C3	1.388 (5)	C12—C13	1.364 (6)
C2—C14	1.494 (5)	C12—H12	0.93
C3—C4	1.376 (6)	C13—H13	0.93
C3—H3	0.93	C14—H14A	0.96
C4—C5	1.368 (5)	C14—H14B	0.96
C4—H4	0.93	C14—H14C	0.96
C5—C6	1.375 (5)	C15—H15A	0.96
C5—H5	0.93	C15—H15B	0.96
C6—H6	0.93	C15—H15C	0.96
			
O2—S1—O1	117.85 (15)	C13—C8—C9	119.3 (3)
O2—S1—N1	109.70 (15)	C13—C8—C7	117.2 (3)
O1—S1—N1	103.55 (14)	C9—C8—C7	123.4 (3)
O2—S1—C1	109.46 (14)	C10—C9—C8	120.7 (4)
O1—S1—C1	109.93 (14)	C10—C9—H9	119.7
N1—S1—C1	105.55 (14)	C8—C9—H9	119.7
C7—N1—S1	122.6 (2)	C11—C10—C9	118.2 (4)
C7—N1—H1N	128 (3)	C11—C10—C15	121.4 (4)
S1—N1—H1N	109 (3)	C9—C10—C15	120.4 (4)
C6—C1—C2	121.9 (3)	C10—C11—C12	122.4 (4)
C6—C1—S1	116.3 (2)	C10—C11—H11	118.8
C2—C1—S1	121.9 (2)	C12—C11—H11	118.8
C3—C2—C1	115.8 (3)	C13—C12—C11	119.0 (4)
C3—C2—C14	119.3 (3)	C13—C12—H12	120.5
C1—C2—C14	124.9 (3)	C11—C12—H12	120.5
C4—C3—C2	122.7 (3)	C12—C13—C8	120.3 (4)
C4—C3—H3	118.6	C12—C13—H13	119.8
C2—C3—H3	118.6	C8—C13—H13	119.8
C5—C4—C3	120.3 (3)	C2—C14—H14A	109.5
C5—C4—H4	119.9	C2—C14—H14B	109.5
C3—C4—H4	119.9	H14A—C14—H14B	109.5
C4—C5—C6	119.3 (3)	C2—C14—H14C	109.5
C4—C5—H5	120.3	H14A—C14—H14C	109.5
C6—C5—H5	120.3	H14B—C14—H14C	109.5
C5—C6—C1	120.0 (3)	C10—C15—H15A	109.5
C5—C6—H6	120.0	C10—C15—H15B	109.5
C1—C6—H6	120.0	H15A—C15—H15B	109.5
O3—C7—N1	120.2 (3)	C10—C15—H15C	109.5
O3—C7—C8	122.8 (3)	H15A—C15—H15C	109.5
N1—C7—C8	117.0 (3)	H15B—C15—H15C	109.5
			
O2—S1—N1—C7	51.6 (3)	C2—C1—C6—C5	−1.1 (5)
O1—S1—N1—C7	178.3 (3)	S1—C1—C6—C5	177.6 (3)
C1—S1—N1—C7	−66.2 (3)	S1—N1—C7—O3	−1.1 (5)
O2—S1—C1—C6	−7.5 (3)	S1—N1—C7—C8	179.1 (2)
O1—S1—C1—C6	−138.4 (2)	O3—C7—C8—C13	−20.9 (5)
N1—S1—C1—C6	110.5 (2)	N1—C7—C8—C13	158.9 (3)
O2—S1—C1—C2	171.3 (2)	O3—C7—C8—C9	157.4 (3)
O1—S1—C1—C2	40.4 (3)	N1—C7—C8—C9	−22.8 (5)
N1—S1—C1—C2	−70.7 (3)	C13—C8—C9—C10	2.1 (5)
C6—C1—C2—C3	0.8 (5)	C7—C8—C9—C10	−176.3 (3)
S1—C1—C2—C3	−178.0 (2)	C8—C9—C10—C11	−1.5 (6)
C6—C1—C2—C14	179.1 (3)	C8—C9—C10—C15	177.8 (4)
S1—C1—C2—C14	0.4 (5)	C9—C10—C11—C12	−1.6 (8)
C1—C2—C3—C4	0.0 (5)	C15—C10—C11—C12	179.1 (5)
C14—C2—C3—C4	−178.5 (4)	C10—C11—C12—C13	4.1 (9)
C2—C3—C4—C5	−0.3 (6)	C11—C12—C13—C8	−3.3 (8)
C3—C4—C5—C6	0.0 (6)	C9—C8—C13—C12	0.4 (6)
C4—C5—C6—C1	0.7 (5)	C7—C8—C13—C12	178.8 (4)

### Hydrogen-bond geometry (Å, °)

**Table:**

*D*—H···*A*	*D*—H	H···*A*	*D*···*A*	*D*—H···*A*
N1—H1N···O1^i^	0.83 (2)	2.06 (2)	2.884 (4)	179 (4)

Symmetry codes: (i) −*x*+1, −*y*+1, −*z*+1.
